# Hormone replacement therapy in women with cancer and risk of cancer-specific mortality and cardiovascular disease: a protocol for a cohort study from Scotland and Wales

**DOI:** 10.1186/s12885-021-08065-3

**Published:** 2021-03-24

**Authors:** Úna McMenamin, Blánaid Hicks, Carmel Hughes, Peter Murchie, Julia Hippisley-Cox, Tom Ranger, Carol Coupland, Chris Cardwell

**Affiliations:** 1 Centre for Public Health, ICSB, Queen’s University Belfast, Royal Victoria Hospital, Belfast, Ireland BT12 6BA; 2 School of Pharmacy, Queen’s University Belfast, Belfast, Northern Ireland; 3 Division of Applied Health Sciences Section, Academic Primary Care, Foresterhill, Aberdeen, UK; 4 Primary Care Epidemiology, Nuffield Department of Primary Care Health Sciences, Medical Sciences Division, University of Oxford, Oxford, UK; 5 Division of Primary Care, University of Nottingham, Nottingham, UK

**Keywords:** Cancer, Hormone replacement therapy, Mortality, Cardiovascular disease

## Abstract

**Background:**

Hormone replacement therapy (HRT) is widely used and has proven benefits for women with menopausal symptoms. An increasing number of women with cancer experience menopausal symptoms but the safety of HRT use in women with cancer is unclear. There are particular concerns that HRT could accelerate cancer progression in women with cancer, and also that HRT could increase the risk of cardiovascular disease in such women. Therefore, our primary aim is to determine whether HRT use alters the risk of cancer-specific mortality in women with a range of common cancers. Our secondary objectives are to investigate whether HRT alters the risk of second cancers, cardiovascular disease, venous thromboembolism and all-cause mortality.

**Methods:**

The study will utilise independent population-based data from Wales using the SAIL databank and Scotland based upon the national Prescribing Information System. The study will include women newly diagnosed with common cancers from 2000 to 2016, identified from cancer registries. Women with breast cancers will be excluded. HRT will be ascertained using electronic prescribing in Wales or dispensing records in Scotland. The primary outcome will be time to cancer-specific mortality from national mortality records. Time-dependent cox regression models will be used to calculate hazard ratios (HR) and 95% confidence intervals (95% CIs) for cancer specific death in HRT users compared with non-users after cancer diagnosis after adjusting for relevant confounders, stratified by cancer site. Analysis will be repeated investigating the impact of HRT use immediately before cancer diagnosis. Secondary analyses will be conducted on the risk of second cancers, cardiovascular disease, venous thromboembolism and all-cause mortality. Analyses will be conducted within each cohort and pooled across cohorts.

**Discussion:**

Our study will provide evidence to inform guidance given to women diagnosed with cancer on the safety of HRT use and/or guide modifications to clinical practice.

## Background

Menopausal hormone replacement therapy (HRT) is widely used globally [1] and has been shown to reduce menopausal related vasomotor symptoms (hot flashes, flushing, and night sweats) and urogenital atrophy [2]. HRT preserves bone mass and is approved by the Food and Drug Administration to prevent postmenopausal osteoporosis [3]. It has also been shown to reduce joint pain, mood swings, sleep disturbances and improve quality of life [2]. Consequently, it has been acknowledged that a reluctance to treat menopausal symptoms can cause unnecessary suffering [4, 5].

### Use of HRT in women with cancer

An increasing number of women with cancer experience menopausal symptoms. HRT is contraindicated in patients with breast cancer and oestrogen-dependent cancers [3, 6, 7] but not in patients with other cancers and authors have argued against the denial of HRT in these patients except when based upon evidence, given the benefits of HRT [8]. Numerous researchers have made recommendations, based upon limited evidence, for and against the use of HRT in patients with cancer depending upon the site of their cancer [8–10].

### HRT and cancer-specific mortality

Various studies have provided evidence suggesting that oestrogen may accelerate the progression of certain cancers. For instance, preclinical studies suggest that oestrogen stimulates growth in bladder [11] and gastric cancer cell lines [12] and lung cancer mouse models [13]. Also, observational studies have shown increases in the risk of glioma and meningioma with use of oestrogen only HRT [14] and the Women’s Health Initiative trial showed a marked increase in death from lung cancer in the oestrogen plus progestin group compared with placebo (HR 1.71, 95%CI 1.16 2.52) [15]. Despite this suggestive evidence, there have not yet been any epidemiological studies of HRT use after bladder, gastric or brain cancer and survival. Although three epidemiological studies have investigated HRT use before lung cancer diagnosis and survival, their results were conflicting [16–18] and none investigated HRT use after diagnosis.

The role of oestrogen in cancer is complex, however, and there is contrasting evidence that oestrogen could delay cancer progression at several cancer sites. For instance, preclinical studies have shown that oestrogen inhibits cell growth and/or induces apoptosis in oesophageal adenocarcinoma [19] and malignant melanoma cell lines [20]. HRT use is associated with a reduced risk of various cancers including liver [21], non-Hodgkin lymphoma [22], and colorectal cancer [23]. A number of studies have investigated hormone replacement therapy after colorectal cancer [24, 25] and melanoma [26, 27] but epidemiological evidence at other cancer sites is sparse. For instance, HRT before diagnosis was associated with reduced mortality in 234 patients with primary liver cancer [21], but HRT after diagnosis was not investigated and in a study of 130 leukaemia patients no excess recurrences were observed in HRT users [28].

### HRT and cardiovascular disease

Studies in the general population have observed that women taking HRT have increased risks of various cardiovascular diseases including coronary heart disease [29], stroke [30] and venous thromboembolism (VTE) [31], particularly in the first years of use [29]. VTE is of particular concern as cancer patients are already at increased risk [32] and it is a common cause of death in cancer patients [33]. Despite this evidence, the risk of cardiovascular disease, and VTE, in cancer patients using HRT has not been investigated previously in large epidemiological cohorts.

### Aims

Our primary aim is to determine the association between HRT use and the risk of cancer-specific mortality in women with a range of common cancers. Our secondary aims are to determine the association between HRT and risk of all-cause mortality, second cancers, cardiovascular disease and VTE in women with cancer.

## Methods/design

### Data sources

The study will utilise the Scottish National Prescribing Information System (Scotland) [34] and the SAIL databank (Wales) [35]. Data sources were selected because they have high quality cancer registry data linked to mortality and prescribing or dispensing data (see Table 1 for details).

**Table 1:** Characteristics of included cohorts

Country	Cancer data source	Diagnosis years	Medication data source	Outcome data source	End follow-up	Outcome
Wales (SAIL databank)	Welsh Cancer Intelligence and Surveillance Unit (National Cancer registry for Wales)	2000 to 2016	Primary Care GP dataset (GP prescription records)	Annual District Death Extract	2019	Cancer-specific death
Scotland (eDRIS)	Scottish Cancer Registry	2009 to 2016	Prescribing Information System (dispensed medications)	National Records of Scotland Death Records	2019	Cancer-specific death

### Cohorts

Population-based cohorts of newly diagnosed, from 2000 to 2016, incident female cancer patients aged 40 to 79 will be identified within each data source. The eighteen most common female cancers, excluding breast cancer (as HRT is generally contraindicated in breast cancer patients), will be investigated including: lung, colorectal, cervical, uterine, malignant melanoma, ovarian, non-Hodgkin’s lymphoma, pancreas, kidney, leukaemia, oral, bladder, oesophagus, thyroid, multiple myeloma, brain, gastric and liver. Patients previously diagnosed with other invasive cancer diagnoses (apart from non-melanoma skin cancer) will be excluded.

### Exposure

HRT use will be ascertained from electronic general practitioner (GP) prescribing records (Wales) or dispensing records (Scotland). We will capture both the route of administration (oral, transdermal, vaginal, injection or implant) and type of HRT including oestrogen-only (conjugated equine oestrogen and estradiol) and combined oestrogen with progestogen preparations (oestrogen with medroxyprogesterone acetate, dydrogesterone, norethisterone acetate, norgestrel/levonorgestrel, or drospirenone).

### Outcome

The primary outcome will be cancer-specific mortality (based upon cancer as the underlying cause of death) from national mortality records (see Table 1). Secondary outcomes of all-cause mortality (from mortality records), any second cancer (and by type, from cancer registry records), and cardiovascular disease (from hospital admissions data or mortality records) including separately coronary heart disease, stroke, VTE and any cardiovascular disease (based upon coronary heart disease or stroke), will also be investigated.

### Covariates

The following covariates will be identified: age, year, ethnicity, stage (from cancer registry records), cancer treatment (including radiotherapy, chemotherapy and surgery, from cancer registry records), grade (from cancer registry records), relevant comorbidities (including Charlson comorbidity conditions, from hospital admissions in Scotland and GP records and hospital admissions in Wales), medication use (including aspirin, statins and other medications associated with cancer survival), hysterectomy/oophorectomy (from hospital admissions in Scotland and hospital admissions and GP records in Wales), family history of cancer (from GP records, not available in Scotland), deprivation (based upon the relevant Index of Multiple Deprivation [34, 35]) and smoking and Body Mass Index (BMI from GP records, not available in Scotland).

### Statistical analysis

Two main analyses will be conducted investigating HRT use after diagnosis and immediately before diagnosis.

In the analysis of HRT use after diagnosis, patients will be followed from 6 months after cancer diagnosis to cancer-specific mortality (censored on death from other causes or end of follow-up). Consequently, patients who died from any cause in the first 6 months after cancer diagnosis will be excluded as it seems unlikely that HRT use after diagnosis could impact upon such deaths (in sensitivity analyses this duration will be varied, described later). HRT use will be modelled as a time varying covariate to avoid immortal time bias [36], i.e. patients will be considered non-users and then users after a lag of 6 months after their first HRT prescription. A lag is recommended in studies of medication use and cancer survival [37] as it moves the exposed period forward which is more likely to reflect when the medication will may have an impact. A dose-response analysis will be conducted by duration of use based upon daily defined doses (DDDs) and number of prescriptions. An individual will be considered a non-user prior to 6 months after first HRT use, a user of 0 to 1 year of prescriptions from 6 months after first prescription to 6 months after their 12th prescription (or 365th DDD) and a greater user after this time. Time-dependent cox regression models will be used to calculate hazard ratios (HRs), and 95% confidence intervals (CIs), for HRT use after cancer diagnosis adjusting for age, year, ethnicity, stage, cancer treatment, grade, comorbidities, prior hysterectomy/oophorectomy, medication use (as time-varying covariates) and deprivation. A two-stage analysis procedure using random effects models will be conducted to pool results across cohorts [38]. Separate analyses will be conducted by type and route of administration of HRT.

In the analysis of HRT before diagnosis, cancer patients will be followed from diagnosis to cancer-specific mortality. HRT use will be identified in the 18 months before diagnosis excluding HRT use in the six months immediately before diagnosis, as depending upon the cancer drug use can increase in this period due to cancer symptoms [39]. Cox regression models will be used to calculate HRs and 95% CIs for cancer-specific death in HRT users compared with non-users. Age, year, ethnicity, comorbidities, prior hysterectomy/oophorectomy and medication use before diagnosis will be included in these cox regression models to calculate adjusted HRs and 95%CIs. Stage, grade and cancer treatments will be excluded from the models investigating HRT before diagnosis, as these could lie on the causal pathway. As before two-stage methods will pool results across cohorts.

A number of additional analyses will be conducted. First, the main analysis will be repeated additionally adjusting for characteristics not available in both cohorts e.g. family history, smoking and BMI (only available in Welsh data). An analysis will be conducted restricted to women over age 55 (i.e. post-menopausal women) and an analysis will be conducted widening the age groups to include younger women aged 18 to 40 (Scottish data). An analysis of HRT after diagnosis will be conducted using a new-user design i.e. restricting analyses to cancer patients who did not use HRT before diagnosis [40], and after adjustment for HRT before diagnosis. A separate analysis will be conducted investigating the timing of HRT initiation after cancer diagnosis. Analyses of HRT after diagnosis will be conducted varying the lag from 6 months to 1 and 2 years.

The primary analysis strategy will be repeated for the secondary outcome of all-cause mortality and second cancers. A different analysis strategy will be used to investigate the secondary outcome of cardiovascular disease and VTE. In these analyses current use of hormone replacement therapy after cancer diagnosis will be modelled as a time-varying covariate. Patients will become a current user upon the date of each hormone replacement therapy prescription, and remain a user for the duration of the prescription, based upon the daily defined dose, plus 30 days to account for any residual effect. The exposure period from the end of one prescription plus 30 days to the start of the next will be allocated to past use. Cox regression models will then be used to calculate HRs and 95% CI for cardiovascular disease overall and by type comparing hormone replacement therapy current use and past use to non-users, adjusting for the confounders mentioned previously.

In general, multiple imputation will be used to impute missing values particularly for stage, smoking and BMI. As recommended cancer-specific death status and cumulative hazard will be included in imputation models [41], along with exposure and confounder variables and results will be combined using Rubin’s rules. STATA 16 will be used for all analyses.

### Sample size calculation

Sample size calculations were conducted based upon the number of cancer cases in published studies from Scotland [42, 43], and in Wales based upon published counts of cases from the Welsh Cancer Intelligence and Surveillance Unit assuming 90% have GP linked data (provided by SAIL databank). The number of deaths was estimated based upon survival at 6 months and 5 years from English published female survival curves [44]. In sample size calculations we estimated that 7% of female cancer patients would use HRT after diagnosis similar to published reports (e.g. 5% of a Swedish colorectal cancer cohort used HRT after diagnosis [25], and 9% of a Danish melanoma cohort used HRT after diagnosis [26]).

For instance, we estimate there will be 22,000 colorectal cancer cases diagnosed in Scotland (diagnosed 2009 to 2016) and Wales (diagnosed 2000 to 2016), of whom, approximately 20% (4400) will die within 6 months (and be removed from analysis of HRT after diagnosis) and a further 31% (6820) will die within 5 years. Therefore, based on a cohort of 17,600 colorectal cancer patients and 6820 cancer-specific deaths, we would have over 80% power to detect, as significant at the 5% level, a HR for cancer-specific mortality of 1.15 (or 0.85) for HRT users after diagnosis (i.e. we could detect an increased risk of cancer-specific mortality in HRT users of 15%) using Schoenfeld’s method [45]. Similarly, in gastric cancer patients, we anticipate 3300 patients and 1181 cancer-specific deaths in the analysis of HRT after diagnosis and therefore we would have over 80% power to detect, as significant at the 5% level, a HR for cancer-specific mortality of 1.45 for HRT users after diagnosis. A similar analysis is being conducted in England using QResearch and we anticipate pooling results across the three cohorts which will further increase power.

## Discussion

These large population cohort studies from Scotland and Wales will provide real world data on the association between HRT use and the risk of cancer-specific mortality in women with a range of common cancers. These cohorts will also provide information on the risk of other potential adverse outcomes of HRT use in cancer patients, such as cardiovascular disease, VTE, second cancers and all-cause mortality. This study will provide cancer patients and clinicians with evidence on the safety of HRT in cancer patients, allowing them to make better informed decisions on whether to use HRT. The study will also provide important mechanistic insights into the role of oestrogen in cancer progression.

## Data Availability

Not applicable.

## Abbreviations

BMI: body mass index
CI: confidence interval
DDD: daily defined dose
GP: general practitioner
HRT: hormone replacement therapy
HR: hazard ratio
VTE: venous thromboembolism

## References

[1] Ameye L, Antoine C, Paesmans M, De Azambuja E, Rozenberg S (2014). Menopausal hormone therapy use in 17 European countries during the last decade. Maturitas..

[2] Baber RJ, Panay N, Fenton A (2016). 2016 IMS recommendations on womens midlife health and menopause hormone therapy. Climacteric..

[3] Goodman NF, Cobin RH, Ginzburg SB, Katz IA, Woode DE (2011). American Association of Clinical Endocrinologists. American Association of Clinical Endocrinologists Medical Guidelines for Clinical Practice for the diagnosis and treatment of menopause. Endocr Pract.

[4] Manson JE, Kaunitz AM (2016). Menopause management — getting clinical care Back on track. N Engl J Med.

[5] Newson LR (2016). Best practice for HRT: unpicking the evidence. Br J Gen Pract.

[6] Cobin RH, Goodman NF (2017). American Association of Clinical Endocrinologists and American College of endocrinology position statement on menopause–2017 update. Endocr Pract.

[7] Joint Formulary Committee (2018). British National Formulary.

[8] Deli T, Orosz M, Jakab A. Hormone Replacement Therapy in Cancer Survivors – Review of the Literature. Pathol Oncol Res. 2019. 10.1007/s12253-018-00569-x PMC7109141 30617760

[9] Biglia M, Gadducci A, Ponzone R, Roagna R, Sismondi P (2004). Hormone replacement therapy in cancer survivors. Maturitas..

[10] Kuhle CL, Kapoor E, Sood R, Thielen JM, Jatoi A, Faubion SS (2016). Menopausal hormone therapy in cancer survivors: a narrative review of the literature. Maturitas..

[11] Shen SS, Smith CL, Hsieh JT, Yu J, Kim IY, Jian W, et al. Expression of estrogen receptors-α and -β in bladder cancer cell lines and human bladder tumor tissue. Cancer. 2006;106(12):2610–6. 10.1002/cncr.21945. 10.1002/cncr.21945 16700038

[12] Wang X, Chen Q, Huang X, Zou F, Fu Z, Chen Y, et al. Effects of 17β-estradiol and tamoxifen on gastric cancer cell proliferation and apoptosis and ER-α36 expression. Oncol Lett. 2017;13(1):57–62. 10.3892/ol.2016.5424. 10.3892/ol.2016.5424 PMC5244966 28123522

[13] Hammoud Z, Tan B, Badve S, Bigsby RM (2008). Estrogen promotes tumor progression in a genetically defined mouse model of lung adenocarcinoma. Endocr Relat Cancer.

[14] Benson VS, Kirichek O, Beral V, Green J (2015). Menopausal hormone therapy and central nervous system tumor risk: large UK prospective study and meta-analysis. Int J Cancer.

[15] Chlebowski RT, Schwartz AG, Wakelee H, Anderson GL, Stefanick ML, Manson JE, et al. Women's Health Initiative investigators. Oestrogen plus progestin and lung cancer in postmenopausal women (Women’s health initiative trial): a post-hoc analysis of a randomised controlled trial. Lancet. 2009;374(9697):1243–51. 10.1016/S0140-6736(09)61526-9. 10.1016/S0140-6736(09)61526-9 PMC2995490 19767090

[16] Clague J, Reynolds P, Henderson KD, Sullivan-Halley J, Ma H, Lacey JV, et al. Menopausal hormone therapy and lung cancer-specific mortality following diagnosis: The California teachers study. PLoS One. 2014;9(7). 10.1371/journal.pone.0103735 PMC4117568 25079077

[17] Ganti AK, Sahmoun AE, Panwalkar AW, Tendulkar KK, Potti A (2006). Hormone replacement therapy is associated with decreased survival in women with lung cancer. J Clin Oncol.

[18] Huang B, Carloss H, Wyatt SW, Riley E (2009). Hormone replacement therapy and survival in lung cancer in postmenopausal women in a rural population. Cancer..

[19] Sukocheva OA, Wee C, Ansar A, Hussey DJ, Watson DI (2013). Effect of estrogen on growth and apoptosis in esophageal adenocarcinoma cells. Dis Esophagus.

[20] Kanda N, Watanabe S (2001). 17Β-estradiol, progesterone, and Dihydrotestosterone suppress the growth of human melanoma by inhibiting Interleukin-8 production. J Invest Dermatol.

[21] Hassan MM, Botrus G, Abdel-Wahab R, Wolff RA, Li D, Tweardy D, et al. Estrogen replacement reduces risk and increases survival times of women with hepatocellular carcinoma. Clin Gastroenterol Hepatol. 2017;15(11):1791–9. 10.1016/j.cgh.2017.05.036. 10.1016/j.cgh.2017.05.036 PMC5901750 28579181

[22] Kane EV, Bernstein L, Bracci PM, Cerhan JR, Costas L, Dal Maso L, et al. Postmenopausal hormone therapy and non-hodgkin lymphoma: a pooled analysis of interlymph case-control studies. Ann Oncol. 2013;24(2):433–41. 10.1093/annonc/mds340. 10.1093/annonc/mds340 PMC3551484 22967995

[23] Grodstein F, Newcomb PA, Stampfer MJ (1999). Postmenopausal hormone therapy and the risk of colorectal cancer: a review and meta-analysis. Am J Med.

[24] Chan JA, Meyerhardt JA, Chan AT, Giovannucci EL, Colditz GA, Fuchs CS (2006). Hormone replacement therapy and survival after colorectal cancer diagnosis. J Clin Oncol.

[25] Ji J, Sundquist J, Sundquist K (2018). Use of hormone replacement therapy improves the prognosis in patients with colorectal cancer: a population-based study in Sweden. Int J Cancer.

[26] Hicks BM, Kristensen KB, Pedersen SA, Holmich LR, Pottegard A (2019). Hormone replacement therapy and the risk of melanoma in post-menopausal women. Hum Reprod.

[27] MacKie RM, Bray CA (2004). Hormone replacement therapy after surgery for stage 1 or 2 cutaneous melanoma. Br J Cancer.

[28] Yang X, Wang C, He X, Wei J, Wang Y, Li X, et al. Hormone therapy for premature ovarian insufficiency patients with malignant hematologic diseases. Climacteric. 2017;20(3):268–73. 10.1080/13697137.2017.1309382. 10.1080/13697137.2017.1309382 28399661

[29] Vandenbroucke JP (2009). The HRT controversy: observational studies and RCTs fall in line. Lancet..

[30] Prentice RL, Langer RD, Stefanick ML, Howard BV, Pettinger M, Anderson GL, et al. Combined analysis of Women's Health Initiative observational and clinical trial data on postmenopausal hormone treatment and cardiovascular disease. Am J Epidemiol. 2006;163(7):589–99. 10.1093/aje/kwj079. 10.1093/aje/kwj079 16484450

[31] Vinogradova Y, Coupland C, Hippisley-Cox J. Use of hormone replacement therapy and risk of venous thromboembolism: nested case-control studies using the QResearch and CPRD databases. BMJ. 2019;364. 10.1136/bmj.k4810 PMC6326068 30626577

[32] Khalil J, Bensaid B, Elkacemi H, Afif M, Bensaid Y, Kebdani T, et al. Venous thromboembolism in cancer patients: an underestimated major health problem. World J Surg Oncol. 2015;13(1):204. 10.1186/s12957-015-0592-8. 10.1186/s12957-015-0592-8 PMC4486121 26092573

[33] Khorana AA (2010). Venous thromboembolism and prognosis in cancer. Thromb Res.

[34] Alvarez-Madrazo S, McTaggart S, Nangle C, Nicholson E, Bennie M (2016). Data resource profile: the Scottish National Prescribing Information System (PIS). Int J Epidemiol.

[35] Ford DV, Jones KH, Verplancke JP, Lyons RA, John G, Brown G, et al. The SAIL databank: building a national architecture for e-health research and evaluation. BMC Health Serv Res. 2009;9(1):157. 10.1186/1472-6963-9-157. 10.1186/1472-6963-9-157 PMC2744675 19732426

[36] Levesque LE, Hanley JA, Kezouh A, Suissa S (2010). Problem of immortal time bias in cohort studies: example using statins for preventing progression of diabetes. BMJ..

[37] Chubak J, Boudreau DM, Wirtz HS, McKnight B, Weiss NS (2013). Threats to validity of nonrandomized studies of postdiagnosis exposures on cancer recurrence and survival. J Natl Cancer Inst.

[38] Stukel TA, Demidenko E, Dykes J, Karagas MR (2001). Two-stage methods for the analysis of pooled data. Stat Med.

[39] Pottegård A, Hallas J (2017). New use of prescription drugs prior to a cancer diagnosis. Pharmacoepidemiol Drug Saf.

[40] Ray WA (2003). Evaluating medication effects outside of clinical trials: new-user designs. Am J Epidemiol.

[41] White IR, Royston P (2009). Imputing missing covariate values for the Cox model. Stat Med.

[42] Gray RT, Coleman HG, Hughes C, Murray LJ, Cardwell CR. Low-dose aspirin use and survival in colorectal cancer: Results from a population-based cohort study. BMC Cancer. 2018;18(1). 10.1186/s12885-018-4142-y PMC6389196 29486728

[43] Spence AD, Busby J, Johnston BT, Baron JA, Hughes CM, Coleman HG, et al. Low-dose aspirin use does not increase survival in 2 independent population-based cohorts of patients with esophageal or gastric cancer. Gastroenterology. 2018;154(4):849–860.e1. 10.1053/j.gastro.2017.10.044 29122547

[44] Rachet B, Woods LM, Mitry E, Riga M, Cooper N, Quinn MJ, et al. Cancer survival in England and Wales at the end of the 20th century. Br J Cancer. 2008;99(S1):S2–S10. 10.1038/sj.bjc.6604571. 10.1038/sj.bjc.6604571 PMC2557545 18813248

[45] Schoenfeld DA (1983). Sample-size formula for the proportional-hazards regression model. Biometrics..

